# Reinterpretation of the rod-and-frame illusion: a virtual reality study

**DOI:** 10.3389/fnins.2025.1639864

**Published:** 2025-08-12

**Authors:** Michał Adamski, Miroslaw Latka

**Affiliations:** Department of Biomedical Engineering, Wroclaw University of Science and Technology, Wroclaw, Poland

**Keywords:** Rod-and-Frame Test, visual field dependence, multisensory integration, sensory reweighting, virtual reality

## Abstract

**Introduction:**

In the Rod-and-Frame Test (RFT), participants align a pivoted rod with the vertical while viewing a tilted, coaxially mounted frame. In doing so, they can use the edge of the frame and its imaginary diagonal as visual cues. Relying on one of these cues leads to the RFT illusion—an error in determining the vertical. We investigated whether individuals who can use both cues perform more accurately at tilt angles, where errors typically peak.

**Methods:**

Twenty-one young adults completed a Virtual Reality RFT. A bias function was defined to range from 1 (rod rotated consistently toward the edge cue) to –1 (toward the diagonal cue). We calculated the bias for the tilt angles ±35° (where the diagonal cue is visually salient) and alignment errors at ±15° (where errors are high).

**Results:**

The bias and error were strongly correlated (*r* = 0.75). Participants with bias values below –0.5 (indicating reliance on the diagonal cue) at ±15° exhibited errors nearly four times smaller than those with bias values above 0.5 (indicating reliance on the edge cue). For ±35°, the error for such groups was not statistically different.

**Conclusions:**

Reliance on the diagonal cue at large tilt angles (e.g., ±35°) is associated with improved performance at smaller tilt angles (e.g., ±15°). These findings suggest that RFT errors—arising from multisensory integration of visual, vestibular, and proprioceptive inputs—also reflect individual differences in the processing of visual context.

## 1 Introduction

The Rod-and-Frame Test (RFT) setup consists of a rod and a surrounding frame, both of which can independently rotate about the same pivot point. During the test, subjects are asked to align the rod with the true vertical. Nearly 80 years ago, Asch and Witkin discovered the rod-and-frame effect: when the frame is tilted, participants tend to rotate the rod past vertical, in the direction of the frame's tilt (Witkin and Asch, 1948).

The perception of verticality results from the complex integration of visual (Böhmer and Mast, 1999; Hansson et al., 2010; Dockheer et al., 2018), vestibular (Clarke et al., 2003; Pavlou et al., 2003; Kumagami et al., 2009; Hansson et al., 2010; Dockheer et al., 2018), and proprioceptive cues (Anastasopoulos et al., 1999; Trousselard et al., 2003; Barra et al., 2010). Individuals differ in how they weight these sensory inputs: field-dependent individuals, who rely more heavily on visual information to assess body orientation, tend to make larger errors. In contrast, field-independent individuals can suppress misleading visual cues and typically perform better in the RFT (Bednarek and Orzechowski, 2008).

Degeneration of vestibular hair cells and neurons is a typical manifestation of aging that can be observed as early as the fifth decade of life (Zalewski, 2015). Impairment of the otolith organs leads to increased noise in the afferent signals transmitted to the sensory integration centers of the brain. The central nervous system compensates by increasing the weighting of visual inputs in determining the vertical (Lee, 2017a,b). Such sensory reweighting (Curthoys, 2000; Peterka, 2002; Peterka and Loughlin, 2004) contributes to the heightened visual field dependence observed in older adults. Consequently, the RFT has been used not only to characterize cognitive style but also to investigate age-related changes in multisensory integration (Alberts et al., 2019).

(Witkin and Asch 1948) carried out their experiment in a darkened room using a rod and frame covered with fluorescent paint. For several decades, various RFT implementations mimicked the original setup (Erdos, 1979; Zoccolotti et al., 1993; Li and Matin, 2005; Tjernström et al., 2019). In more recent studies, the test has been projected onto a wall (Tasseel-Ponche et al., 2017), implemented using the video eye glasses (Bagust, 2005), displayed on a computer monitor (Razzak et al., 2018), or presented in virtual reality (Bringoux et al., 2009; Adamski et al., 2021; Willey and Liu, 2022; Fujimoto and Ashida, 2022).

The implementation of the Rod-and-Frame Test in a virtual reality environment (VR-RFT) offers significant methodological and practical advantages over traditional 2D or mechanical setups. By its very nature, VR-RFT is reproducible, portable, free of unwanted visual cues, and does not require controlled lighting conditions. Moreover, the immersive VR environment can mimic the visual conditions of the real world, increasing the relevance of the findings in cognitive and perceptual research (Reger et al., 2003). This is why in our research we used a VR-RFT.

In the RFT, subjects can use the edge of the frame and its imaginary diagonal as visual cues (Beh et al., 1971). Relying on one of these cues leads to the RFT illusion—an error in determining the vertical. In this study, we investigate whether individuals who can use both cues perform more accurately at tilt angles, where errors typically peak. To this end, we introduce the concept of RFT bias. Such a function, for a given frame tilt, indicates whether a subject rotates the rod toward one of the cues. We employ the variability of the bias across the set of angles (e.g., clockwise or counterclockwise), termed RFT flexibility, to assess the extent to which the subjects use both visual cues to determine vertical.

## 2 Subjects and methods

### 2.1 Subjects

The research protocol was approved by the Ethics Committee of the Wrocław University of Science and Technology and conducted in accordance with the Declaration of Helsinki. Twenty-one young Polish nationals (12 women, nine men), aged 19–29 years (*M* = 22, *SD* = 2.7), voluntarily participated in the study and provided written informed consent. All participants self-identified as White, were native Polish speakers, and were fluent in English. They were recruited from undergraduate and graduate programs in science, engineering, and mathematics. They did not report any vestibular diseases.

For seven individuals with no prior exposure to virtual reality, a brief familiarization session was conducted before data collection, which included exploring a neutral virtual environment and practicing with the handheld controllers.

### 2.2 VR implementation of the rod and frame test

The experiment was conducted using an *Oculus Quest 3* virtual reality headset running a custom-built Rod-and-Frame Test application developed in *Unity 3D* (version 2021.3) with the *OpenXR* backend. The virtual scene (Supplementary Figure S1) consisted of a white corridor measuring 1.8 × 1.8 units in cross section and 10 units in length. One unit corresponds to ~1 m. The virtual camera, representing the participant's point of view, was located in the center of the corridor.

A black rod modeled as a vertically stretched capsule with a length of 1.3 units was placed five units in front of the camera and attached to the back wall. The corridor was symmetrically but non-uniformly illuminated with point light sources to enhance visual realism and depth perception. For brevity, we will refer to the back wall as the frame since it plays the role of the frame in the classical RFT setup. We want to point out the oversimplification inherent in such nomenclature. The frame edges are also the borders of the wall that the subject looks at. Consequently, the illuminated cuboid corridor also contributes to the sensory conflict experienced by the subjects during the RFT.

Participants used the right-hand controller's thumbstick and/or the buttons to rotate the rod in 0.5° increments. Once the desired alignment was achieved, the left-hand controller was used to confirm the rod's orientation, triggering a reset and advancing to the next trial. The rod's final orientation relative to the virtual gravitational vertical was recorded automatically upon confirmation, using the object's local rotation angle, sampled through Unity's transform component. After confirmation, the rod and corridor smoothly returned to the neutral orientation (0°) over a duration of 1 second. This was followed by a rapid spinning animation of the corridor—a full 360° rotation in both directions—before settling at the next frame orientation. The rod's initial orientation was randomized for each trial. We used 18 frame tilt angles ranging from −40° to +45° in 5° increments, with positive angles corresponding to clockwise rotations.

### 2.3 Study protocol

The experiment consisted of 10 segments. In half of them, participants performed five alignments for frame tilts θ of ±10°, ±20°, ±30°, and ±40°. In the other half, a different set of tilt angles was used: 0°, ±5°, ±15°, ±25°, ±35°, and 45°. The presentation order of θ for a given segment type was determined randomly before the experiment and was identical for all subjects. For example, in the first type of segment, there were a total of 40 trials (five trials for each of the eight tilt angles). To determine the presentation order, we created a list of length 40 containing repeated tilt values [−40, −40, −40, −40, −40, −30, …, 40, 40, 40, 40, 40]. Then, this list was randomized to generate the tilt sequence that was used for each subject. The two lists used in the experiment are provided in the Supplementary material.

The segment types were alternated. To minimize fatigue, a mandatory 5-min break was introduced between segments, which lasted ~10–20 min, depending on the individual's pace. Participants were asked to complete as many segments as they could before experiencing fatigue or loss of concentration, at which point the session was ended. The experiment was carried out over two to four morning sessions, with a total test duration of ~3–5 h per subject. Twenty five trials were collected for each tilt angle.

During testing, participants were seated in a chair and asked to lean against a backrest to maintain a stable and consistent posture. A seated position was chosen due to the length and cognitive demands of the experimental protocol, ensuring that upright stance would not limit task performance or participant endurance.

Before the start of the experiment and at the beginning of each session, participants received verbal instructions in their native language. They were asked to align a virtual rod with the direction of gravity. The instructions were as follows:

“*Your task is to rotate the rod so that it aligns with the vertical—that is, the direction gravity pulls straight downward. Imagine the rod is hanging freely from its upper end, like a plumb line. Try to ignore the tilted frame. Focus only on what you believe is the direction of gravity in the real world.”*

No time constraints were imposed on the alignment task.

### 2.4 Data analysis

#### 2.4.1 RFT bias

The edges of the frame and the imaginary diagonals can serve as visual cues that the subject can use to determine the vertical. For small frame tilts, subjects tend to position the rod toward the rotated edge of the frame. The sign of error *e*_*i*_(θ_*k*_) is the same as the sign of θ_*k*_ (Supplementary Figure S2). (Abdul Razzak and Bagust 2022) refer to such a strategy as the *direct* effect. The alternative strategy–aligning with the diagonal–would result in a much larger error. However, positioning the rod in the direction of the diagonal could be beneficial for large frame tilts. In this case, θ_*k*_ and *e*_*i*_(θ_*k*_) have opposite signs. Following the terminology of (Abdul Razzak and Bagust 2022), we refer to this choice as an *indirect* effect.

To determine whether, for a given frame tilt θ_*k*_, a subject in *N*_*t*_ trials rotates the rod on average toward the edge of the tilted frame or away from it, in other words, whether the direct or indirect effect is observed, we define the RFT bias:


(1) $$\begin{array}{l} b\left(\theta_{k}\right)=\frac{S_{k}^{\left(\text{direct}\right)}-S_{k}^{\left(\text{indirect}\right)}}{N_{t}}, \end{array}$$

where


(2) $$\begin{array}{l} S_{k}^{\left(\text{direct}\right)}=|\left\{i:\text{sign}\left(e_{i}\left(\theta_{k}\right)\right)=\text{sign}\left(\theta_{k}\right)\right\}|, \end{array}$$

(3) $$\begin{array}{l} S_{k}^{\left(\text{indirect}\right)}=|\left\{i:\text{sign}\left(e_{i}\left(\theta_{k}\right)\right)=-\text{sign}\left(\theta_{k}\right)\right\}|. \end{array}$$

In Equations 2, 3 |·| denotes the cardinality of the set.

The bias *b* can vary between −1 and 1. When only a direct effect is observed, the function takes the value of 1. In contrast, a value of −1 indicates an indirect effect.

#### 2.4.2 RFT flexibility

Bias *b*(θ_*k*_) reflects the subject's tendency to align the rod with the frame's edges or diagonals at a given tilt angle θ_*k*_, where θ_*k*_ ∈ Θ. To assess whether subjects change their perceptual strategy across different frame tilt conditions Θ, we define RFT *flexibility* as the interquartile range (IQR) of the set of biases:


(4) $$\begin{array}{l} f=\text{IQR}\left({\left\{b\left(\theta_{k}\right)\right\}}_{\theta_{k}\in \Theta }\right)=Q_{3}-Q_{1}, \end{array}$$

where *Q*_1_ and *Q*_3_ denote the first and third quartiles, respectively.

The interquartile range (IQR) was selected as a measure of variability due to the inherent characteristics of RFT bias—namely, the potential for outliers (e.g., isolated switches at extreme tilt angles) and the frequent occurrence of non-normal distributions in bounded variables. These characteristics make alternative measures such as the range or standard deviation less appropriate.

We calculate flexibility *f* for all nonzero frame tilt angles and separately for clockwise (*f*_*R*_) and counterclockwise (*f*_*L*_) tilt angles.

We also define an flexibility asymmetry index (α_*f*_) as:


(5) $$\begin{array}{l} \alpha_{f}=\frac{f_{R}-f_{L}}{f_{R}+f_{L}}. \end{array}$$

#### 2.4.3 RFT error metrics and error asymmetry index

Let ${\left\{e_{i}\left(\theta_{k}\right)\right\}}_{i=1}^{N_{t}}$ be the set of errors a subject makes when determining the vertical for a given frame tilt θ_*k*_ in *N*_*t*_ trials. We will use the absolute value of the median ẽ(θ_*k*_) of such a set as one of the error metrics:


(6) $$E(\theta_{k})=\left|\tilde{e}(\theta_{k})\right|.$$

The others are the averages of *E*(θ_*k*_) over clockwise (R)


(7) $$\begin{array}{l} E_{R}=\frac{1}{8}\underset{\theta_{k}>0}{\sum }E\left(\theta_{k}\right) \end{array}$$

and counterclockwise (L)


(8) $$\begin{array}{l} E_{L}=\frac{1}{8}\underset{\theta_{k}<0}{\sum }E\left(\theta_{k}\right) \end{array}$$

tilts.

We quantify the asymmetry of the subject's error curve ẽ(θ_*k*_) using the following index:


(9) $$\begin{array}{l} \alpha_{e}=\frac{\sum_{\theta_{k}>0}E\left(\theta_{k}\right)-\sum_{\theta_{k}<0}E\left(\theta_{k}\right)}{\overset{N_{k}}{\underset{k=1}{\sum }}E\left(\theta_{k}\right)}. \end{array}$$

The asymmetry index α_*e*_ ranges from −1 to 1, with positive values indicating that the magnitude of the errors is greater for positive (clockwise) frame tilts. In our experiment, *N*_*k*_ = 16, as we exclude θ = 0° and θ = 45°; the latter is omitted due to the symmetry of the VR scene.

### 2.5 Statistical analysis

All statistical comparisons were performed using a permutation test (Phillip Good, 2005), which is appropriate for the small, non-Gaussian samples analyzed in this study. We used the Python *scipy.stats* implementation, specifically the *permutation_test* function. For each test, 100,000 permutations were generated to estimate the null distribution.

The Spearman correlation coefficient was calculated using the *spearmanr* function from the *scipy.stats* module.

A significance level of 0.05 was used for all statistical tests.

## 3 Results

**Figure 1:**
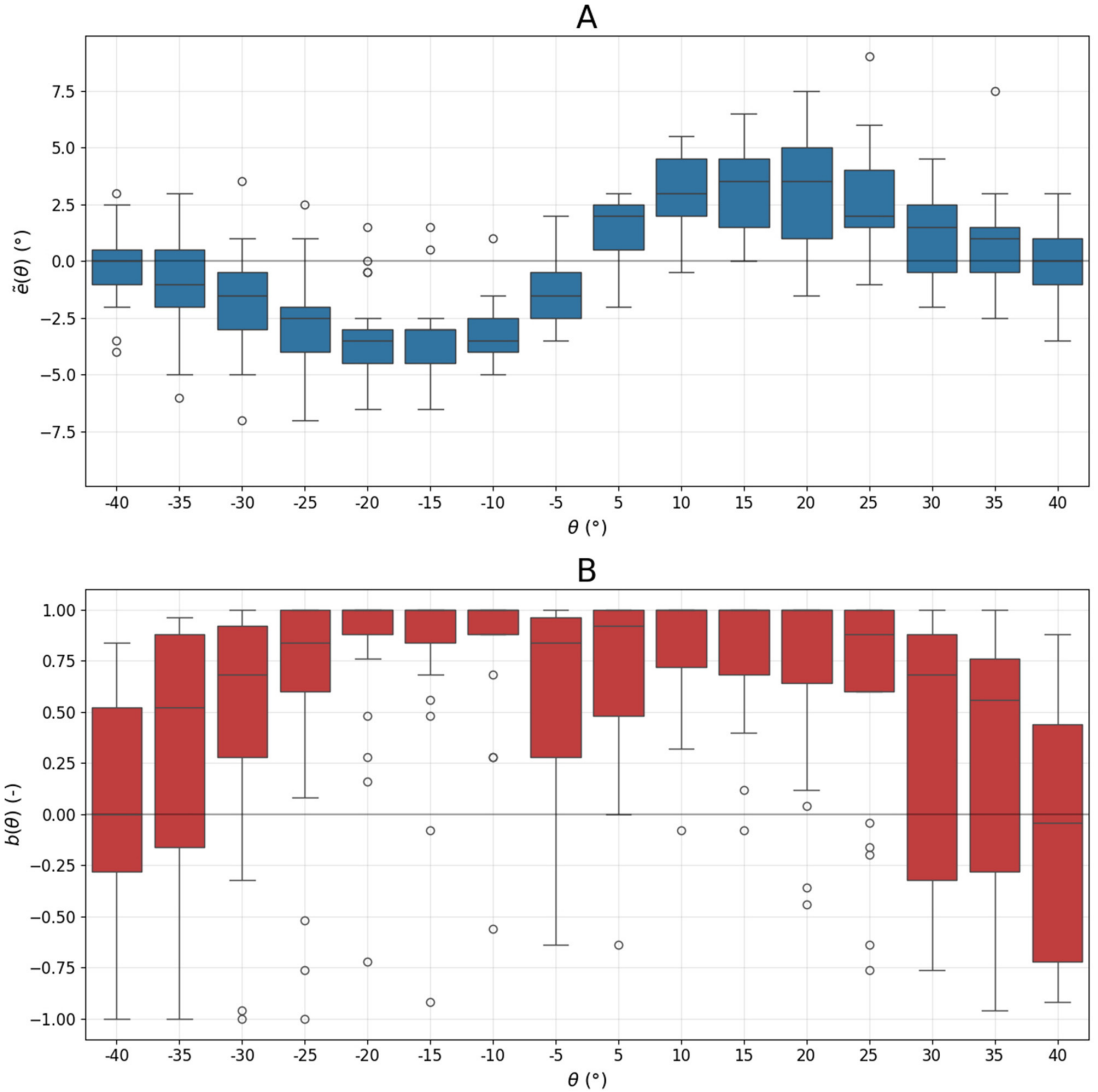
Distribution of the median alignment error **(A)** and bias **(B)** as a function of frame tilt angle θ.

For each subject, we calculated the median alignment error across the 18 frame tilt angles θ used in the experiment. Figure 1A shows the distribution of these medians for the cohort. Although the maximum median error of 3.5° occurs at a tilt of 20° and 15°, the errors between 10° and 20° are similar. A comparable pattern is observed for negative (counterclockwise) θ, where maximum median error occurs at 20° and 10°.

The group-averaged bias is highest in the intervals [−20, −10] and [10, 20], indicating that most subjects exhibit the direct effect—rotating the rod in the direction of the tilted edges of the frame (Figure 1B). For these intervals, the median bias is 1.00. Bias values are notably lower for larger tilts: [−40, −30] and [30, 40] (0.52 and 0.40 respectively), suggesting that some participants rotate the rod toward the frame's diagonal (indirect effect).

**Figure 2:**
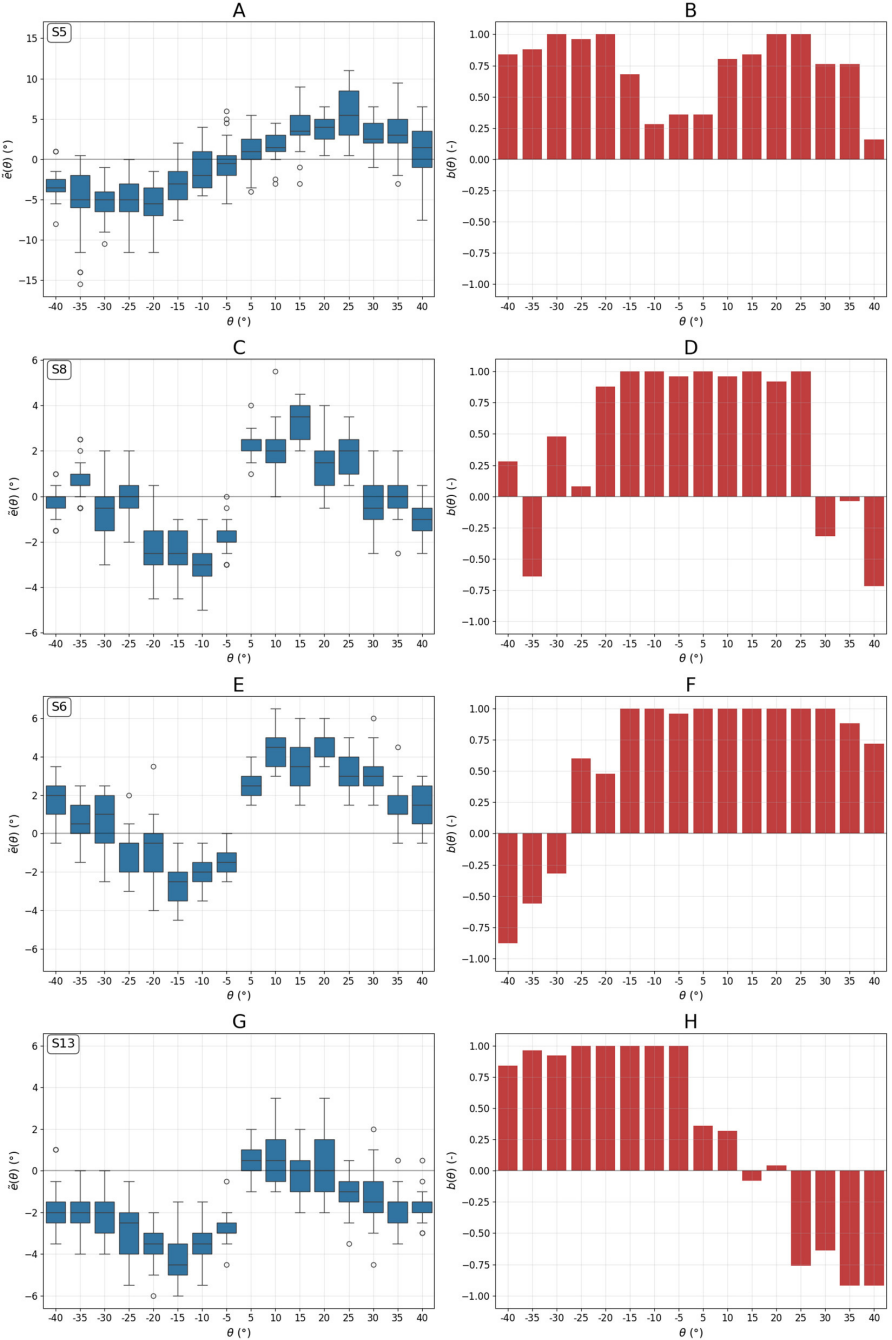
Examples of distribution of RFT alignment error **(A, C, E, G)** and the corresponding bias **(B, D, F, H)** as a function of frame tilt angle θ. The data is organized by subject: the first row shows Subject S5, the second row Subject S8, the third row Subject S6, and the fourth row Subject S13. Panels **(A)** and **(C)** show rather symmetric error curves, in contrast to the asymmetric angular dependence seen in **(E)** and **(G)**. Panel **(B)** shows the bias for the subject who predominantly exhibits only the direct RFT effect. In contrast, panel **(D)** shows that Subject S8 exhibits both direct and indirect effects. For Subjects S6 and S13, the indirect effect is observed only for counterclockwise **(F)** and clockwise **(H)** rotations, respectively.

The group-averaged error curve ẽ(θ) shown in Figure 1A appears fairly symmetric. However, this symmetry may be misleading, as it results from averaging both symmetric (e.g., Figures 2A, C) and asymmetric (e.g., Figures 2E, G) individual error curves.

The error asymmetry index for subjects S5 (−0.13) and S8 (0.08) in Figures 2A, C is low. Subject S5 shows only the direct effect, while subject S8 also displays the indirect effect at large frame tilts.

The error curves for subjects S6 and S13 are clearly asymmetrical, as reflected in their error asymmetry index values (0.39 and −0.56). Subject S6 exhibits the indirect effect only for negative tilt angles, whereas subject S13 shows it primarily for positive tilts. These two cases shed light on how visual cue usage relates to RFT alignment error. For subject S6, the mean absolute error for positive θ is 129% higher than for negative angles (3° vs. 1.31°), with markedly lower RFT flexibility (0.03 vs. 1.35). For subject S13, the error is 256% higher for negative θ (2.88° vs. 0.81°), again with much lower flexibility (0.05 vs. 0.91).

**Figure 3:**
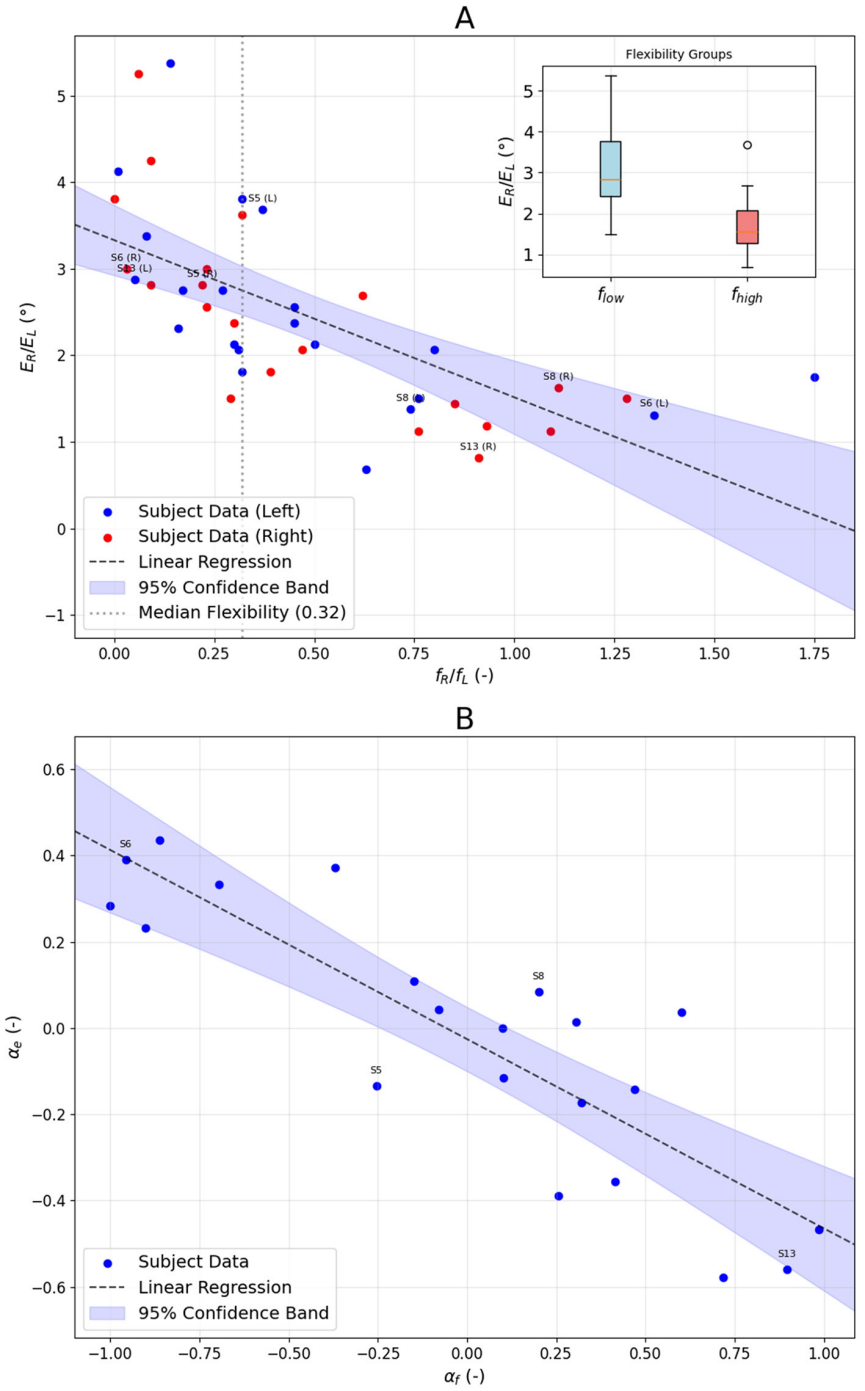
**(A)** Alignment error *E* and flexibility index *f* were calculated for all subjects (S1–S21), separately for clockwise (*R*) and counterclockwise (*L*) frame tilts. The plot illustrates a strong correlation between error and flexibility. We divided the cases into two groups using the median of *f* = 0.32. The inset shows the distribution of *E* for such groups. **(B)** RFT asymmetry index (α_*e*_) and flexibility asymmetry index (α_*f*_) for all subjects (S1–S21). The labels in both subplots were drawn next to the data points corresponding to the case studies presented in Figure 2.

Considering asymmetry in the case studies, we calculated *E*_*R*_ and *E*_*L*_ and the corresponding *f*_*R*_ and *f*_*L*_ (Supplementary Table S1). Such 42 pairs (two for each subject) are shown (Figure 3A). The correlation between error and flexibility was strong: ρ = −0.80 with *p* < 1 × 10^−5^. A linear fit to all data points gave *E*(*f*) = −1.82*f*+3.33, with 16 out of 42 points (38.1%) within the 95% confidence band. To further verify that the flexibility is related to the error in the RFT, we divided the cases into two groups using the median of *f* = 0.32 (Supplementary Table S2). The inset in Figure 3A shows the distribution of *E* for such groups. The median of *E* is equal to 2.84° and 1.56° for low (*f* < 0.32) and high (*f*≥0.32) flexibility, respectively. The percent change of 82% is statistically significant (*p* = 6 × 10^−3^). Figure 3B illustrates the relationship between error asymmetry α_*e*_ and flexibility asymmetry α_*f*_. The Spearman correlation coefficient was ρ = −0.85 with *p* = 3 × 10^−5^. The linear fit yielded α_*e*_ = −0.44α_*f*_−0.03, with 11 out of 21 points (52.4%) within the 95% confidence band.

**Figure 4:**
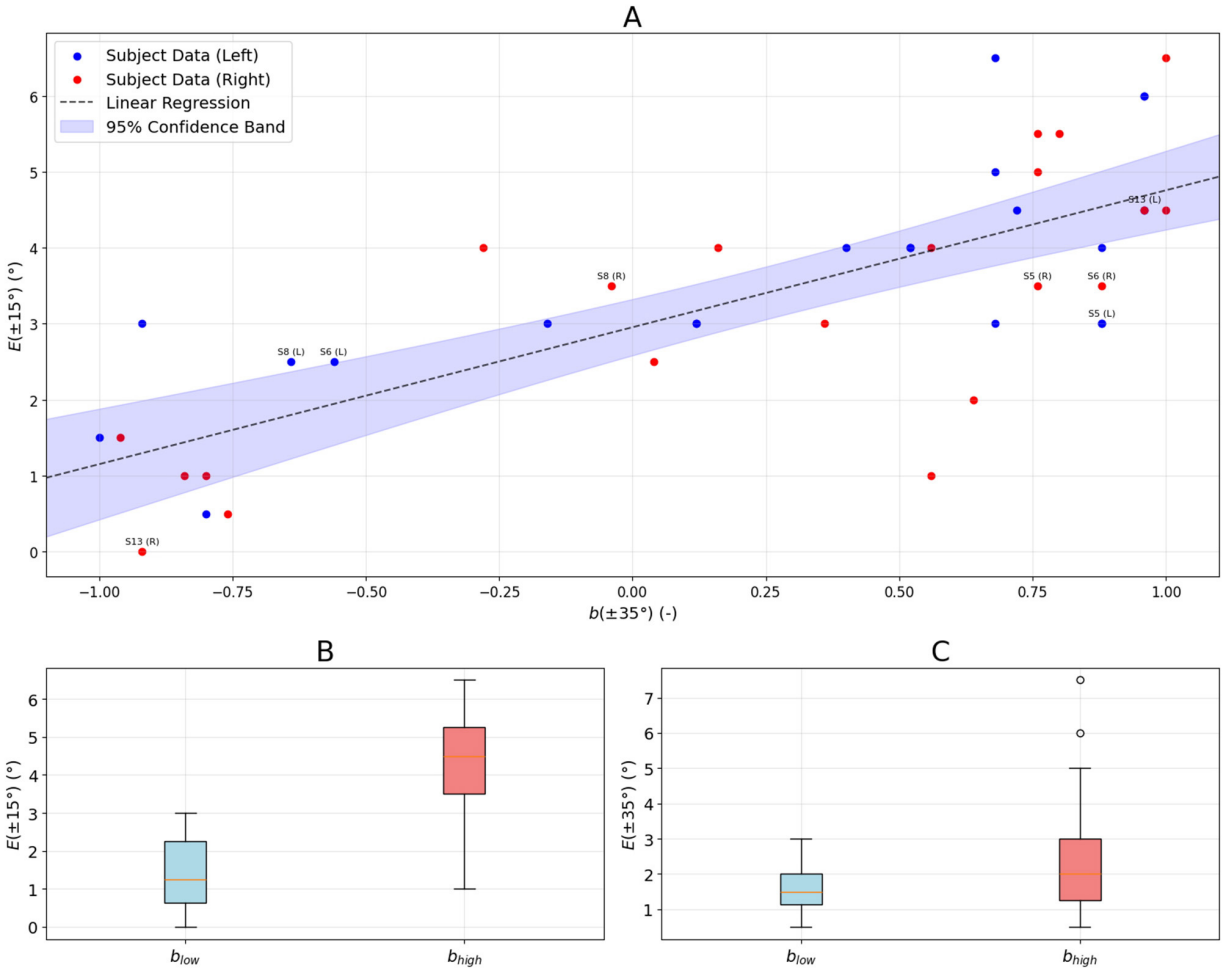
To determine whether subjects who use the frame's diagonal while searching for the vertical are more accurate, we calculated the bias function *b*(θ) at θ = 35° where such a cue is visually salient and the error *E*(θ) at θ = 15° where the group-averaged error curve reaches maximum. Similarly, the calculations were repeated for the corresponding negative tilt angles. The points in **(A)** represent such pairs. The labels were drawn next to the data points corresponding to the case studies presented in Figure 2. **(B)** shows the distribution of errors for low-bias cases (*b* ≤ −0.5, indicating reliance on the diagonal cue) and high-bias cases (*b* ≥0.5, indicating reliance on the edge cue). **(C)** shows the error made by the groups shown in **(B)** at θ = 35°.

To determine whether subjects who use the frame's diagonal while searching for the vertical are more accurate, we calculated the bias function *b*(θ) at θ = 35° where such a cue is visually salient, and the error *E*(θ) at θ = 15° where the group-averaged error curve reaches maximum. Similarly, the calculations were repeated for the corresponding negative tilt angles. We collect results in Supplementary Table S3 and present the spread of these points in Figure 4A. We can see that the higher the bias, the higher the error. The correlation between them is strong ρ = 0.75 with *p* < 1 × 10^−4^. The best linear model was *E*(*b*) = −1.80*b*+2.95. In general, 15 of 42 data points (35.7%) were within the 95% confidence band. Figure 4B shows the distribution of errors for low-bias cases (*b* ≤ −0.5, indicating reliance on the diagonal cue) and high-bias cases (*b* ≥0.5, indicating reliance on the edge cue). The median *E* in the low-bias group was almost four times smaller than that in the high-bias group (1.25° vs. 4.50°, *p* = 0.003). As seen in Figure 4C there was no statistically significant difference in error *E* made by both groups at θ = ±35° (1.50° vs. 2.00°, *p* = 1).

## 4 Discussion

The RFT is a classic paradigm for studying visual field dependence (Witkin and Asch, 1948). Historically, performance on this test has been interpreted primarily in terms of susceptibility to the frame-induced illusion, assumed to reflect a stable individual trait—visual field dependence (Witkin and Asch, 1948; Nair et al., 2018; Abdul Razzak et al., 2020; Abdul Razzak and Bagust, 2022). However, this view may oversimplify the perceptual and cognitive strategies involved. Specifically, participants are not limited to a single visual cue; both the edges of the tilted frame and its diagonals can serve as reference axes (Beh et al., 1971). The diagonals of a tilted square frame can approximate the gravitational vertical, particularly at larger tilt angles. Thus, the selection and weighting of visual cues may influence RFT performance beyond general field dependence.

At small values of θ, the frame's edges play a critical role in the rod alignment strategy. They cause the subject to rotate the rod past the true vertical, as illustrated in Figure 2A for subject S5. The alignment error remains much smaller than the frame's tilt angle (i.e., the maximum possible RFT error), reflecting the multisensory nature of vertical perception. As θ increases, so does the sensory conflict. Due to the frame's symmetry, at θ = 22.5°, neither the edges nor the diagonals are reliable cues for verticality. The saturation and eventual decrease of alignment error at larger tilt angles may reflect a shift in reliance from visual to non-visual (vestibular and proprioceptive) inputs, though this interpretation requires further investigation.

On average, subject S5 exhibits the classic direct effect—rotating the rod in the direction of the frame's tilt—as indicated by a high positive bias value (Figure 2B). In contrast, at θ = 40° and θ = −35°, subject S8 (Figure 2D) rotates the rod opposite to the frame's tilt, toward one of the diagonals, consistent with the indirect effect. The errors *E*(θ) for S8 are substantially smaller than those of S5 (Supplementary Table S2). This raises the question of whether utilizing the diagonal as a visual cue might be advantageous for reducing alignment error. At first glance, this hypothesis may appear controversial, since both the direct and indirect effects contribute to RFT error, as illustrated by subject S6 at θ = ±40° (Figures 2E, F). Nevertheless, Figures 2E, G reveal that RFT errors tend to be smaller on the side of the tilt spectrum where the indirect effect is observed. Similar effect was observed by (Abdul Razzak and Bagust 2022) in a study that used two frame tilts (±18°). A plausible explanation is that subjects' ability to visualize the frame's diagonals reflects their capacity to perceive the visual context in a way that mitigates the RFT illusion at intermediate tilt angles, where alignment errors are typically largest.

Figure 3A shows strong negative correlation ρ = −0.80 with *p* < 1 × 10^−5^ between mean absolute error and RFT flexibility. Moreover, the error *E* for the low-flexibility group is 84% higher than that of the high-flexibility group. One can see in Figure 3B that the asymmetry in RFT flexibility mirrors the asymmetry in alignment error (the correlation coefficient ρ = −0.85 with *p* = 1 × 10^−5^), further supporting the notion that an individual's cue-use strategy is closely tied to task performance. The most compelling evidence that the ability to use the reconstructed frame's diagonal to determine the vertical underlies good performance at tilt angles where the RFT error is highest is provided by data presented in Figure 4. We can see that subjects who relied on the diagonal to determine the vertical at ±35° (for such angles, this cue is visually salient), at ±15° made the error almost four times smaller than those who relied on the edge. It should be emphasized that the error that both groups made at ±35° was not statistically different.

The presented findings suggest that RFT errors—arising from multisensory (visual, vestibular, proprioceptive) integration—also reflect an individual's propensity for cue integration, which attenuates the sensitivity to the edge cue.

Accurate perception of verticality at intermediate tilt angles may be particularly important for maintaining postural control and balance in natural environments, especially for populations with impaired vestibular function or in aging, where reliance on visual cues may increase (Agathos et al., 2015).

A significant question is whether RFT flexibility in visual cue use is a fixed individual characteristic or a malleable skill. Future studies could investigate whether individuals can be trained to improve their flexibility—for example, through explicit instruction or targeted practice—and whether such training translates to better RFT performance and, potentially, to improvements in other real-world spatial orientation tasks (Willey and Jackson, 2014) and balance control. The review of (Cortés-Pérez et al. 2021) shows the examples of such line of research that involve both immersive and non-immersive VR.

Several limitations are inherent to the present study.

- Sample size and composition: the study was conducted with a relatively small sample of 21 young adults. To ensure generalizability, future studies should involve larger and more demographically diverse samples.
- Age-related differences: the longitudinal study by (Bagust et al. 2013) demonstrated significant changes in RFT during maturation. Older adults may also exhibit different patterns of visual reliance and cue integration (Alberts et al., 2019; Agathos et al., 2015).
- Clinical populations: the study focused on young individuals. Examining patients with vestibular disorders and neurological impairments can provide insight into how these conditions affect visual context processing and RFT performance, for example, in peripheral vestibular disorders (Obrero-Gaitán et al., 2021).
- Head movement during the RFT: the position of the head may significantly influence the RFT error by interacting with visual and proprioceptive signals (Czarnolewski, 2024). There is no conclusive evidence that natural, unforced head movements, such as small tilts or left-to-right shifts, significantly affect the in RFT error and its angular dependence (symmetry). We monitored head position and found no obvious relation between natural head movements and RFT error symmetry; a detailed analysis of this effect will be presented in a follow-up publication.
- Duration of the test session: in our experiment, the subjects self-determined the duration of each session, which can be considered a limitation of the study.

## 5 Conclusions

We revisited the classic Rod and Frame Test (RFT) using a virtual reality implementation. Our findings suggest a need to reinterpret the significance of RFT alignment error: it reflects not only visual field dependence but also individual differences in how visual context is processed. Participants who used both visual cues—the frame's edges and diagonals—were better able to reduce or even overcome the RFT illusion, particularly at tilt angles most relevant to balance control in everyday environments.

## Data Availability

The datasets presented in this study can be found in online repositories. The names of the repository/repositories and accession number(s) can be found below: Mendeley Data 10.17632/bm85znjmw6.2.
