# Supplementary material for: Reinterpretation of the rod-and-frame illusion: a virtual reality study
Source: Front Neurosci. 2025 Aug 12;19:1639864. doi: 10.3389/fnins.2025.1639864 (PMC12379729; doi:10.3389/fnins.2025.1639864)
Supplement: Supplementary file 1 [file Data_Sheet_1.pdf]

Supplementary Material

1 FIGURES

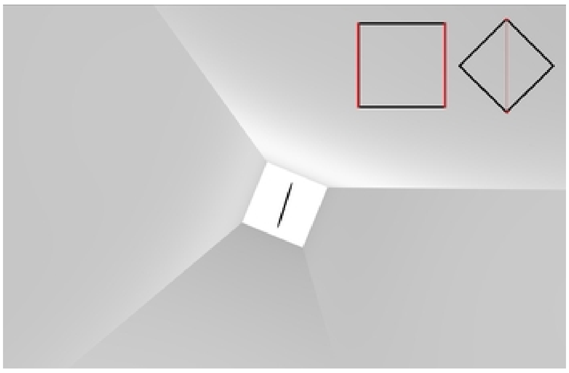

Figure S1. Virtual reality testing room (scene) used in the rod and frame experiment. A subject views a rod of length 1.3 units from a distance of 5 units within a cuboid room with width and height equal to 1.8 units. The room is illuminated by a spotlight positioned behind the subject. The subject determines the subjective visual vertical for 18 randomly presented frame tilt angles ranging from $-40°$ to $45°$ in steps of $5°$. In the upper right corner, the visual cues used to infer verticality are shown: the edges of the frame and one of its diagonals. Note that this image does not reflect the visual experience of the subjects during the test, as it corresponds to a single-eye perspective.

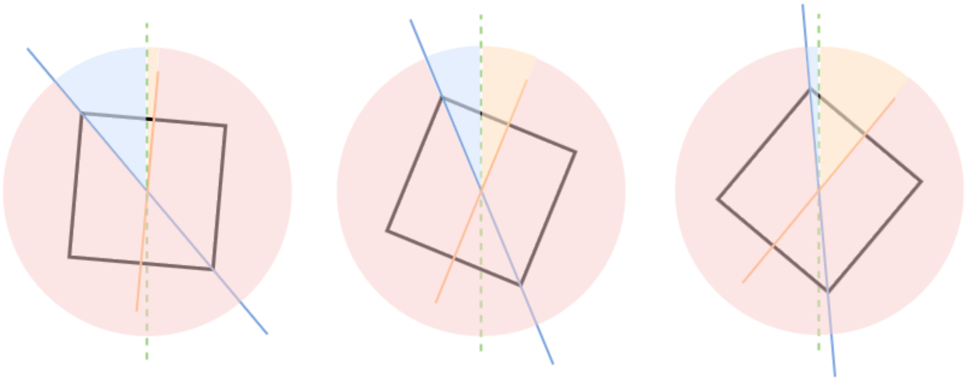

Figure S2. During the Rod and Frame Test (RFT), a subject attempts to align the rod with the vertical. The edges of the frame, represented by the orange line, and the imaginary diagonals (blue) can act as visual cues. For simplicity, we display only one diagonal. With small frame tilts (e.g., $5°$), subjects tend to position the rod toward the rotated frame, resulting in an error within the yellow-shaded arc. In this instance, the sign of the error matches the sign of the frame tilt (direct frame effect). The alternative strategy—aligning with the diagonal—would lead to a much larger error, as shown by the blue-shaded arc. Here, the error's sign is opposite to the frame tilt (indirect frame effect). For a frame tilt of $22.5°$, either strategy would produce a comparable error. However, with a large frame tilt (e.g., $40°$), aligning with the diagonal may be advantageous, as demonstrated by the relative widths of the arcs for the direct and indirect effects.

2 THE ORDER OF TILT PRESENTATION

Order of tilt presentation for segment 1 type:

[30, -20, 10, -20, 20, -40, -20, -40, 30, -30, -40, 40, -30, 30, 40, -30, -10, 40, -40, -40, 10, -30, 40, 10, -20, 20, 30, -20, 40, -10, 30, 20, 20, 10, -10, 10, -30, -10, 20, -10]

Order of tilt presentation for segment 2 type:

[45, 5, -25, -15, -35, 15, 0, 35, 25, -5, 45, 5, -25, -15, -35, 15, 0, 35, 25, -5, 45, 5, -25, -15, -35, 15, 0, 35, 25, -5, 45, 5, -25, -15, -35, 15, 0, 35, 25, -5, 45, 5, -25, -15, -35, 15, 0, 35, 25, -5]

3 TABLES

Table S1. The values of test parameters for all subjects

ID	$E_L[°]$	$E_R[°]$	f_L	f_R	α_e	α_f	$E(\pm 15^{\circ})$	$b(\pm 35^{\circ})$
S1	3.81	2.69	0.32	0.62	-0.17	0.32	4.00	-0.28
S2	1.50	1.19	0.76	0.93	-0.12	0.10	0.50	-0.76
S3	2.38	1.12	0.45	1.09	-0.36	0.42	1.00	-0.80
S4	2.06	2.56	0.31	0.23	0.11	-0.15	4.00	0.56
S5	3.69	2.81	0.37	0.22	-0.13	-0.25	3.50	0.76
S6	1.31	3.00	1.35	0.03	0.39	-0.96	3.50	0.88
S7	2.75	3.00	0.27	0.23	0.04	-0.08	5.50	0.80
S8	1.38	1.62	0.74	1.11	0.08	0.20	3.50	-0.04
S9	2.31	2.38	0.16	0.30	0.01	0.30	4.00	0.16
S10	1.75	2.81	1.75	0.09	0.23	-0.90	4.50	0.96
S11	1.81	1.81	0.32	0.39	0.00	0.10	2.50	0.04
S12	2.56	1.12	0.45	0.76	-0.39	0.26	1.00	0.56
S13	2.88	0.81	0.05	0.91	-0.56	0.90	0.00	-0.92
S14	2.06	5.25	0.80	0.06	0.44	-0.86	6.50	1.00
S15	4.12	1.50	0.01	1.28	-0.47	0.98	1.50	-0.96
S16	0.69	1.50	0.63	0.29	0.37	-0.37	2.00	0.64
S17	5.38	1.44	0.14	0.85	-0.58	0.72	1.00	-0.84
S18	3.38	3.62	0.08	0.32	0.04	0.60	5.50	0.76
S19	2.75	2.06	0.17	0.47	-0.14	0.47	3.00	0.36
S20	2.12	3.81	0.30	0.00	0.28	-1.00	4.50	1.00
S21	2.12	4.25	0.50	0.09	0.33	-0.69	5.00	0.76

Table S2. The values of the error E and flexibility f were calculated for the positive (R) and negative (L) tilt angles. Then such data were divided into low and high flexibility groups based on the median value of the flexibility ($f_{median} = 0.32$). High flexibility group: $f \geq f_{median}$, Low flexibility group: $f < f_{median}$. Such data were used in the inset of Figure 3.

Subject-Side	E (°)	f	Group
S1-L	3.81	0.32	High
S1-R	2.69	0.62	High
S2-L	1.50	0.76	High
S2-R	1.19	0.93	High
S3-L	2.38	0.45	High
S3-R	1.12	1.09	High
S4-L	2.06	0.31	Low
S4-R	2.56	0.23	Low
S5-L	3.69	0.37	High
S5-R	2.81	0.22	Low
S6-L	1.31	1.35	High
S6-R	3.00	0.03	Low
S7-L	2.75	0.27	Low
S7-R	3.00	0.23	Low
S8-L	1.38	0.74	High
S8-R	1.62	1.11	High
S9-L	2.31	0.16	Low
S9-R	2.38	0.30	Low
S10-L	1.75	1.75	High
S10-R	2.81	0.09	Low
S11-L	1.81	0.32	High
S11-R	1.81	0.39	High
S12-L	2.56	0.45	High
S12-R	1.12	0.76	High
S13-L	2.88	0.05	Low
S13-R	0.81	0.91	High
S14-L	2.06	0.80	High
S14-R	5.25	0.06	Low
S15-L	4.12	0.01	Low
S15-R	1.50	1.28	High
S16-L	0.69	0.63	High
S16-R	1.50	0.29	Low
S17-L	5.38	0.14	Low
S17-R	1.44	0.85	High
S18-L	3.38	0.08	Low
S18-R	3.62	0.32	High
S19-L	2.75	0.17	Low
S19-R	2.06	0.47	High
S20-L	2.12	0.30	Low
S20-R	3.81	0.00	Low
S21-L	2.12	0.50	High
S21-R	4.25	0.09	Low

Table S3. The error $E(\pm 15^\circ)$, $E(\pm 35^\circ)$, and the corresponding bias $b(\pm 35^\circ)$. R and L denote the positive and negative tilt angles, respectively. The data were divided into three groups: Diagonal dominant: bias ≤ -0.5, Non dominant: $-0.5 <$ bias < 0.5, Edge dominant: bias ≥ 0.5. The data presented in this Table were used to in Figure 4.

Subject-Side	E(±15°)	E(±35°)	b(±35°)	Group
S1-L	6.50	2.00	0.68	Edge
S1-R	4.00	0.50	-0.28	Non
S2-L	3.00	0.00	0.12	Non
S2-R	0.50	1.50	-0.76	Diagonal
S3-L	4.00	1.00	0.52	Edge
S3-R	1.00	2.00	-0.80	Diagonal
S4-L	3.00	0.50	0.12	Non
S4-R	4.00	1.50	0.56	Edge
S5-L	3.00	5.00	0.88	Edge
S5-R	3.50	3.00	0.76	Edge
S6-L	2.50	0.50	-0.56	Diagonal
S6-R	3.50	1.00	0.88	Edge
S7-L	4.00	1.00	0.52	Edge
S7-R	5.50	1.50	0.80	Edge
S8-L	2.50	0.50	-0.64	Diagonal
S8-R	3.50	0.00	-0.04	Non
S9-L	4.50	1.00	0.72	Edge
S9-R	4.00	0.00	0.16	Non
S10-L	3.00	1.50	-0.92	Diagonal
S10-R	4.50	1.50	0.96	Edge
S11-L	3.00	0.50	0.68	Edge
S11-R	2.50	0.00	0.04	Non
S12-L	3.00	3.00	0.88	Edge
S12-R	1.00	1.50	0.56	Edge
S13-L	4.50	2.00	0.96	Edge
S13-R	0.00	1.50	-0.92	Diagonal
S14-L	3.00	1.00	-0.16	Non
S14-R	6.50	7.50	1.00	Edge
S15-L	6.00	3.50	0.96	Edge
S15-R	1.50	2.00	-0.96	Diagonal
S16-L	0.50	1.00	-0.80	Diagonal
S16-R	2.00	1.00	0.64	Edge
S17-L	6.00	6.00	0.96	Edge
S17-R	1.00	2.50	-0.84	Diagonal
S18-L	5.00	2.50	0.68	Edge
S18-R	5.50	2.50	0.76	Edge
S19-L	4.00	1.50	0.88	Edge
S19-R	3.00	0.50	0.36	Non
S20-L	1.50	3.00	-1.00	Diagonal
S20-R	4.50	3.00	1.00	Edge
S21-L	4.00	1.00	0.40	Non
S21-R	5.00	2.50	0.76	Edge
